# Putative Bifunctional Chorismate Mutase/Prephenate Dehydratase Contributes to the Virulence of *Acidovorax citrulli*

**DOI:** 10.3389/fpls.2020.569552

**Published:** 2020-09-25

**Authors:** Minyoung Kim, Jongchan Lee, Lynn Heo, Sang-Wook Han

**Affiliations:** Department of Plant Science and Technology, Chung-Ang University, Anseong, South Korea

**Keywords:** chorismate mutase/prephenate dehydratase, *Acidovorax citrulli*, virulence, proteomics, biofilm

## Abstract

*Acidovorax citrulli* (*Ac*) is a plant pathogenic bacterium that causes bacterial fruit blotch (BFB) in cucurbit crops. Despite its importance in the cucurbit industry, resistant cultivars/lines against BFB have not yet been identified. Therefore, there is a need to characterize the virulence factors/mechanisms in *Ac* to control the disease. Chorismate mutase, a key enzyme in the shikimate pathway, produces aromatic amino acids. Here, we report the functions of putative bifunctional chorismate mutase/prephenate dehydratase in *Ac* (CmpAc) determined by proteomic analysis and phenotypic assays. *Ac* strain lacking CmpAc, *AcΔcmpAc*(EV), were significantly less virulent on watermelon in the germinated-seed inoculation and leaf infiltration assays. Sequence analysis revealed that CmpAc possesses two distinct domains: chorismate mutase and prephenate dehydratase, indicating that CmpAc is a bifunctional protein. Auxotrophic assays demonstrated that CmpAc is required for the biosynthesis of phenylalanine, but not tyrosine. The comparative proteomic analysis revealed that CmpAc is mostly involved in cell wall/membrane/envelop biogenesis. Furthermore, *AcΔcmpAc*(EV) showed reduced twitching halo production and enhanced biofilm formation. In addition, *AcΔcmpAc*(EV) was less tolerant to osmotic stress but more tolerant to antibiotics (polymyxin B). Thus, our study provides new insights into the functions of a putative bifunctional protein related to virulence in *Ac*.

## Introduction

*Acidovorax citrulli* (*Ac*), formerly known as *A. avenae subsp. citrulli*, is a gram-negative, rod-shaped, and seed-borne bacterium. *Ac* is the causal agent of bacterial fruit blotch (BFB) in watermelon, melon, and other cucurbit crops (Schaad et al., 1978; Willems et al., 1992). Watermelon seedlings infected by *Ac* show water-soaked lesions on cotyledons as initial symptoms, followed by the collapse of the emerging plant and subsequent wilting and dying of the seedlings. In case of the fruit, the infected watermelon shows dark, olive-like lesions. In the later stage, the water-soaked lesion spreads, leading to the ultimate decay and collapse of the fruit flesh by secondary infection or saprophytes (Latin and Hopkins, 1995). Despite the importance of this disease in watermelon production, lines/cultivars of watermelon resistant to *Ac* have not been identified. Thus, there is a limit to the efficient management of BFB, and *Ac* remains a major threat to the worldwide cucurbit crop industry (Burdman and Walcott, 2012). Although *Ac* strains isolated from rockmelon showed similar virulence on watermelon (O’Brien and Martin, 1999), *Ac* is generally known to be divided into two groups: group I strains are severely virulent on melon, and group II strains are mainly virulent on watermelon and moderately virulent on other Cucurbitaceae (Walcott et al., 2000).

Several virulence factors/mechanisms in *Ac* have been characterized. In the previous studies, *Ac* mutants showing impaired twitching motility and biofilm formation were less virulent on melon (Bahar et al., 2009). The type IV pili of *Ac* are also known to be involved in surface adhesion and virulence (Bahar et al., 2010). Deletion of *aacR* and *aacI* genes involved in quorum sensing in *Ac* showed reduced virulence but enhanced biofilm formation (Wang et al., 2016). In addition, Bahar et al. reported that a polar flagellum is involved in the full virulence in melon (Bahar et al., 2011). Similar to other gram-negative plant pathogenic bacteria, regulatory proteins for type III secretion system (T3SS), HrpG and HrpX, are also indispensable for the pathogenicity of *Ac* (Zhang et al., 2018). Recently, diverse type III effectors have been characterized in *Ac* (Jiwenez-Guerrero et al., 2020). In addition, functions of type II and VI secretion systems, quorum sensing mechanisms, and regulators related with ferric uptake have been also characterized regarding the virulence of *Ac* (Burdman and Walcott, 2012; Johnson and Walcott, 2013; Tian et al., 2015; Liu et al., 2019). However, other virulence factors/mechanisms related with biochemical pathways of *Ac* are still poorly understood.

Chorismate mutase is a critical enzyme in the shikimate pathway responsible for the synthesis of aromatic amino acids. This enzyme catalyzes chorismate to produce prephenate, which is an intermediate molecule for the biosynthesis of phenylalanine and tyrosine (Kast et al., 2000). Notably, many bacterial chorismate mutase are bifunctional proteins possessing two catalytic capacities for chorismate mutase and prephenate dehydratase (Duggleby et al., 1978; Ahmad et al., 1988; Huccetogullari et al., 2019). These bifunctional proteins are indispensable for phenylalanine production but not for tyrosine production because prephenate dehydratase is involved in phenylalanine but not tyrosine biosynthesis. The chorismate mutase of *Mycobacterium tuberculosis* plays a crucial role in the pathogenesis of tuberculosis (Khanapur et al., 2017). In addition, genes related to the shikimate pathway are involved in the production of toxoflavin, which is known to be a major virulence factor and promote tolerance to UV in *Burkholderia glumae*, the causative agent of bacterial panicle blight in rice (Karki and Ham, 2014). The secretome analysis from *Xanthomonas citri*. subsp. *citri*, a citrus canker pathogen, revealed chorismate mutase as a potential virulence factor (Ferreira et al., 2016). However, functions of chorismate mutase associated with virulence or other mechanisms have not been reported in *Ac*.

In this study, we report the functions of putative bifunctional chorismate mutase/prephenate dehydratase in the *Ac strain* KACC17005 whose genomic information was previously annotated (Park et al., 2017). Screening of the *Ac* Tn5-insertional library revealed a mutant that had lost virulence and contained a chorismate mutase gene disrupted by Tn5; the protein domain information indicates that it is a putative bifunctional chorismate mutase/prephenate dehydratase in *Ac* (CmpAc). To postulate mechanisms related to CmpAc, a comparative proteomic analysis combined with clusters of orthologous groups (COGs) were employed. Based on the proteomics, phenotypic changes in *AcΔcmpAc* were investigated.

## Materials and Methods

### Bacterial Strains and Growth Conditions

The *Ac* strain KACC17005 belonging to group II (Song et al., 2020) was used in this study as a wild-type strain. *Ac* strains were grown in TSB (Tryptic Soy Broth Soybean-Casein Digested, 30 g/L) or M9 (47.7 mM Na_2_HPO_4_·7H_2_O, 22 mM KH_2_PO_4_, 8.6 mM NaCl, 18.7 mM NH_4_Cl, 2 mM MgSO_4_, 0.1 mM CaCl_2_, 20 ml of 20% glucose in 1 L) at 28°C. The *Escherichia coli* strain DH5α was used for cloning genes and generating constructs. To identify genes disrupted by Tn5, the *E. coli* strain EC100D was used (Lucigen, Madison, WI, USA). *E. coli* strains were cultivated at 37°C in LB (Luria Bertani; 1% tryptone, 0.5% yeast extract, and 1% NaCl) with appropriate antibiotics. For selection, antibiotics were added to the media at the following final concentrations: kanamycin, 50 µg/ml; rifampicin, 50 µg/ml; gentamicin, 10 µg/ml; and ampicillin, 100 µg/ml.

### Selection of *AcΔcmpAc* and Generation of the Complemented Strain

Bacterial strains and plasmids used in this study are listed in **Supplementary Table 1**. The knock out mutant of CmpAc was identified by screening the Tn5 insertional library generated with EZ-Tn5™ <R6Kγori/KAN-2> Insertion Kit (Lucigen, Middleton, WI, USA) as the manufacture’s protocol. Briefly, to construct the Tn5 insertional library, EZ-Tn5 transposome was introduced into the wild-type strain by electroporation using Bio Rad Micropulser™ (Bio-Rad, Hercules, CA, USA) under 2.5 kV. Tn5 insertional mutants were selected on TSA containing kanamycin and rifampicin as a selective marker. Approximately, 4,000 mutants were generated. Each colony was transferred into TSB containing kanamycin and rifampicin in 96-well cell plates, incubated for 2 days at 28°C and kept at −80°C. A germinated-seed inoculation assay was used for selecting virulence-deficient mutants. After extracting genomic DNA from the selected mutant, DNA fragments interrupted by Tn-5 were cloned using the manufacturer’s protocol (Lucigen). The interrupted regions were verified by Sanger sequencing using two primers (KAN-2F, 5′-acctacaacaaagctctcatcaacc-3′, and R6KAN-2R, 5′-ctaccctgtggaacacctactct-3′). The identified mutant was named as *AcΔcmpAc*. To generate the construct for the complemented strain, the open reading frame of *cmpAc* was amplified using *cmpAc*-specific primers: 5′-aagcttatgtcccaatcccc-3′, and 5′-ggatcctcagtggtggtggtggtggtgcggcgccaccg-3′. The amplicon was cloned into pGem-T easy vector (Promega, Madison, WI, USA) to produce pGem-CmpAc plasmid, which was confirmed by Sanger sequencing. The confirmed plasmid was digested with *Hind*III and *BamH*I, and the digested CmpAc construct was cloned again into pBBR1-MCS5 (Kovach et al., 1995), a broad host range vector containing *LacZ* promoter, creating pBBR1-CmpAc plasmid, which was then introduced into *AcΔcmpAc* cell by electroporation using Bio Rad Micropulser™ (Bio-Rad) under 2.5 kV. The transformant was selected on tryptic soy agar (TSA) with kanamycin and gentamycin and confirmed by polymerase chain reaction (PCR) using the *cmpAc*-specific primers used for cloning *cmpAc* amplicon, generating the complemented strain, *AcΔcmpAc*(CmpAc). To minimize the side effect caused by the pBBR1-MCS5 vector, the empty vector was introduced into *Ac* and *AcΔcmpAc* to create *Ac*(EV) and *AcΔcmpAc*(EV), respectively. The transformants were selected on TSA with gentamycin/rifampicin or gentamycin/rifampicin/kanamycin and confirmed by PCR using pBBR1-MCS5 specific primers: 5′-cagggttttcccagtcacga-3′ and 5′-atgcttccggctcgtatgtt-3′.

### Pathogenicity Test

*Citrullus lanatus* var. *vulgaris* line SBA provided by Partner Seed Company (Gimje, Korea) was used for pathogenicity assay, which was carried out by two inoculation methods (the germinated-seed inoculation and the leaf infiltration methods). For germinated-seed inoculation, a previously reported protocol was used with slight modifications (Bahar et al., 2009). To improve the penetration rate, the seeds were germinated on moisturized-filter papers for 2 days. *Ac* strains were grown on TSA at 28°C, suspended in 10 mM MgCl_2_ to an OD_600nm_ of 0.3, which corresponds to approximately 10^8^ colony forming unit (CFU)/ml, and diluted (10^−2^) with the same buffer to obtain 10^6^ CFU/ml. Ten germinated seeds were placed into 50-ml conical tubes containing 20 ml of bacterial suspension and incubated with moderate shaking (100 rpm) for 1 h at 22°C. The inoculated seeds were then sown in a 50-pot tray containing sterilized soil and grown in a growth chamber for 7 days. The disease severity of the inoculated watermelon was evaluated for 7 days. The disease severity was rated on a scale of 0–2 as follows: 0, no symptoms; 1, water-soaked region (Spot); 2, wilt. The disease index was then calculated using the following equation: Disease index = [Normal (numbers of plants)×0 + Spot (numbers of plants)×1 + Wilt (numbers of plants)×2]/Total (numbers of plants). For this assay, 10 biological replicates were examined, and seven independent experiments were carried out. For infiltration, the germinated seeds were grown in a growth chamber for 2 weeks until four true leaves stage. *Ac* strains were grown in the TSA plate and suspended in 10 mM MgCl_2_ to an OD_600nm_ of 0.3 and diluted (10^-3^) with 10 mM MgCl_2_ to obtain 10^5^ CFU/ml. The bacterial suspensions were infiltrated into first and second true leaves using 3-ml needleless syringes. For counting bacterial cell numbers, the infiltrated leaves were punched by cork-borers (0.4 cm in diameter), and two leaf disks were ground in 200 µL sterilized water using tissue grinders. The extracted *Ac* cells were serially diluted and dotted onto the antibiotics-containing TSA and incubated for 2 days at 28°C. Three biological replicates were employed, and seven independent experiments were carried out in this assay.

### Auxotrophic Assay

The bacterial growth of *Ac*(EV), *AcΔcmpAc*(EV), and *AcΔcmpAc*(CmpAc) was evaluated in four different conditions (TSB, M9, M9 with one mM tyrosine, and M9 with one mM phenylalanine). The *Ac* strains were grown on TSA with appropriate antibiotics. The cultured bacterial cells were harvested and washed twice using sterilized water. After washing, the bacterial cells were adjusted to an OD_600nm_ of 0.3, diluted (10^−3^) with TSB to obtain 10^5^ CFU/ml, and grown in the shaking incubator at 28°C. Bacterial growth was measured using a spectrophotometer at OD_600nm_ for 4 days at 12 h intervals. In minimal media, the bacterial suspension was adjusted to an OD_600nm_ of 0.05 (approximately, 1.7×10^7^ CFU/ml) and observed for 7 days at 24 h intervals. For this assay, three biological replicates for each strain were used, and six independent experiments were carried out.

For proteomic analysis, *Ac* and *AcΔcmpAc* were used, and a label-free shotgun comparative proteomics approach was carried out using a previously reported protocol (Park H. J. et al., 2020). Briefly, *Ac* strains were grown in TSB and harvested at an OD_600_ of 0.5 (approximately, 1.7×10^8^ CFU/ml) by centrifugation. The harvested cells were disrupted by an Ultrasonic Processor (Colo Parmer, Vernon Hills, IL, USA). Total proteins were concentrated using trichloroacetic acid precipitation and digested by trypsin. After cleaning the tryptic-digested proteins using the Sep-Pak Vac 1cc tC18 cartridge (Waters, Milford, MA, USA), peptide concentration was determined using a BCA assay kit (Thermo Fisher Scientific, Waltham, MA, USA). Then, 1 μg of each sample (two strains with three biological replicates) was injected into a split-free nano LC (EASY-nLC II; Thermo Fisher Scientific, Bremen, Germany) combined with an LTQ Velos Pro instrument (Thermo Fisher Scientific) by the nanospray ion mode. Tryptic-digested peptides were loaded onto 7.5 cm of MAGIC C18AQ 200A (5 µm) (Michrom BioResources, Auburn, CA, USA) with 300 nl/min flow rate for 420 min under water/acetonitrile gradient using buffer A (water with 0.1% formic acid) and buffer B (100% acetonitrile with 0.1% formic acid). For the mass spectra, six data-dependent MS/MS scans with m/z in the 300–2,000 range of mass were used. Dynamic exclusion was allowed under one repeat, 0.5 min duration, and 3 min exclusion. The ion charge state selection was permitted for 2^+^ and 3^+^. From the full mass scan, six of the most intense ions were chosen.

The procedure for identifying and quantifying proteins and peptides was followed as reported previously except for the database (Park H. J. et al., 2020). The MS/MS spectra were identified by Thermo proteome discoverer (ver. 1.3.0.399) with a SEQUEST search algorithm. Genome information of *Ac* strain KACC17005 (Accession No. CP023687) was used from the National Center for Biotechnology Information. To improve the credibility, the target-decoy strategy was used (Elias and Gygi, 2007). Two missed cleavages were permitted, and the false discovery rate was 0.01. One hundred ppm precursor was set for mass accuracy, and the probability scores were at least 20. The identified proteins by the Thermo Proteome Discoverer were imported again into Scaffold 4 (Proteome Software, Portland, OR, USA), which was used for comparison. Peptide spectrum matches (PSMs) was used for comparative analyses (Choi et al., 2008). PSMs from the proteins were normalized against the total proteins in a sample. Three biological replicates were used, and the average of PSMs from the replicates was calculated for each protein and compared for the identification of proteins that are differentially abundant (over 2-fold) in *Ac* vs. *AcΔcmpAc*. The statistical analysis was performed using student’s t-test (P < 0.05). COG analysis was used for the categorization of the identified proteins (Tatusov et al., 2000).

### Biofilm Formation

For biofilm formation assay, a previously established protocol was used with slight modification (Park et al., 2020). The *Ac* strains were grown for 24 h in TSA, washed, suspended in TSB to an OD_600nm_ of 0.3, and diluted (10^−3^) with TSB (approximately, 10^5^ CFU/ml). The bacterial suspension was incubated in 96-well polyvinyl chloride (PVC) plate at 28°C for 2 days. After incubation, the bacterial supernatant was removed carefully and washed using sterilized water. After washing, the remaining bacterial cells were stained with 0.1% crystal violet for 30 min, washed twice with sterilized water, suspended in 95% ethanol for 20 min, and measured the absorbance at 590 nm with a Spectramax 190 microplate reader (Molecular Devices, Sunnyvale, CA, USA); 20 biological replicates of each strain were assessed, and three independent experiments were carried out.

### Twitching Motility Assay

The twitching motility assay was carried out as previously reported with slight modification (Turnbull and Whitchurch, 2014). The bacterial suspension was adjusted to an O.D._600nm_ of 0.3 and diluted (10^−2^) with sterilized water (approximately, 10^6^ CFU/ml). Then, 5 μl of bacterial suspension was dotted onto TSA containing 0.5% agar and incubated at 28°C for 2 days. The twitching halos and colony diameter were evaluated by the stereoscopic microscope, a LEICA M205C (LEICA, Wetzlar, Germany). For this assay, five biological replicates of each strain were examined and five independent experiments were carried out.

### Tolerance to Osmotic and Antibiotic Stresses

To investigate the effects of CmpAc in stress conditions, the strains were subjected to two stress conditions, namely, osmotic and antibiotic stress. For the osmotic stress, bacterial cell culture with OD_600nm_ of 0.3 (approximately, 10^8^ CFU/ml) was incubated in the presence of 2.5% NaCl at 28°C for 10 min with shaking (280 rpm) and were then serially diluted and dotted onto a TSA with appropriate antibiotics. After incubation for days, viable cell numbers were measured by a colony counting method. Tolerance against antibiotics was examined by supplementing the medium with 0.1 μg/ml polymyxin B at 28°C for two h with shaking (280 rpm). Water was used as a negative control in the assays. To calculate the survivability, the ratio of bacterial numbers in the control to stress conditions was calculated. For each assay, three biological replicates were evaluated, and at least seven independent experiments were carried out.

### Statistical Analysis

The statistical significance was analyzed by one-way analysis of variance with Tukey HSD^ab^ using SPSS 12.0K software (SPSS, Inc., Chicago, IL, USA). A p-value of less than 0.05 was considered as a statistical difference.

## Results

### Identification of *AcΔcmpAc* Strain and Protein Sequence Analysis of CmpAc

**Figure 1:**
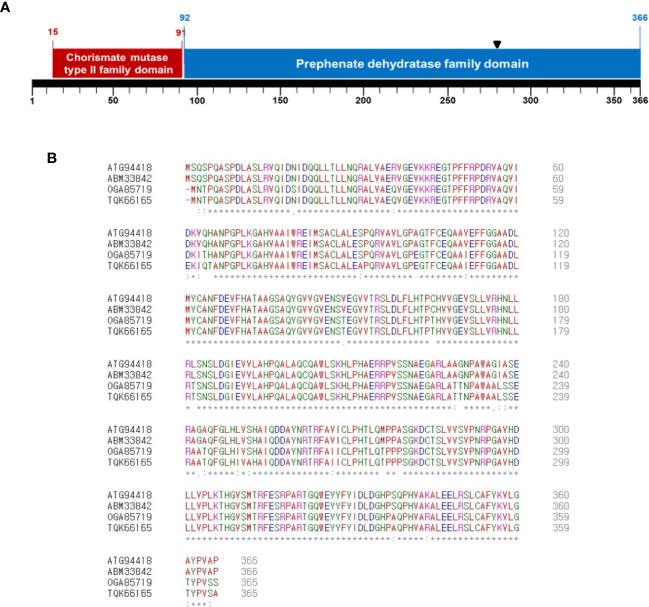
Sequence analysis of deduced amino acids of CmpAc. **(A)** Predicted domains in CmpAc. Red and blue boxes indicate chorismate mutase type II family and prephenate dehydratase family domains, respectively. A black arrow indicates the Tn5 insertional position. **(B)** Sequences alignment of CmpAc (ATG94418), prephenate dehydratase (ABM33842) from *Ac* strain AAC00-1, chorismate mutase (OGA85719) from *Burkholderiales* bacterium GWA2_64_37 (subsurface metagenome), and prephenate dehydratase (TQK66165) from *Nocardioides* sp. SLBN-35 using the Clustal Omega Program. The “*”, “:”, and “.” represent identical residues, conserved substitutions, and semi-conserved substitutions, respectively.

A Tn5-insertional library in the background of *Ac* strain KACC17005 belonging to group II was screened to identify genes involved in the virulence of *Ac*. We found one mutant that did not cause disease on watermelon and confirmed that a gene, which was annotated as chorismate mutase (Accession No. ATG94418; Locus tag, CQB05_10545), was disrupted by Tn5. The deduced amino acids of ATG94418 revealed that the protein possesses two domains: chorismate mutase type II family (15-91 aa) and prephenate dehydratase family (92-366 aa) (**Figure 1A**) , indicating that ATG94418 encodes a putative bifunctional chorismate mutase/prephenate dehydratase protein. In agreement with our prediction, Zhang et al. reported that N-terminal and C-terminal of the P-protein, a bifunctional chorismate mutase/prephenate dehydratase protein, in *E. coli* are required for chorismate mutase and prephenate dehydratase activity, respectively (Zhang et al., 1998). In addition, CmpAc showed high homology with a putative chorismate mutase or prephenate dehydratase in other gram-negative bacteria (**Figure 1B**). ATG94418 showed 100% similarity with a putative prephenate dehydratase (Accession No. ABM33842) in *Ac* strain AAC00-1, 93% similarity with a putative chorismate mutase (Accession No. OGA85719) in *Burkholderiales* GWA2_64_37, and 92% similarity with a putative prephenate dehydratase (Accession No. TQK66165) in *Nocardioides* sp. SLBN-35. This suggests that the bifunctional chorismate mutase/prephenate dehydratase protein is conserved in the genus as well as in other bacteria. Therefore, ATG94418 was named as CmpAc (bifunctional chorismate mutase/prephenate dehydratase in *Ac*).

### CmpAc Is Indispensable for Virulence of *Ac*

**Figure 2:**
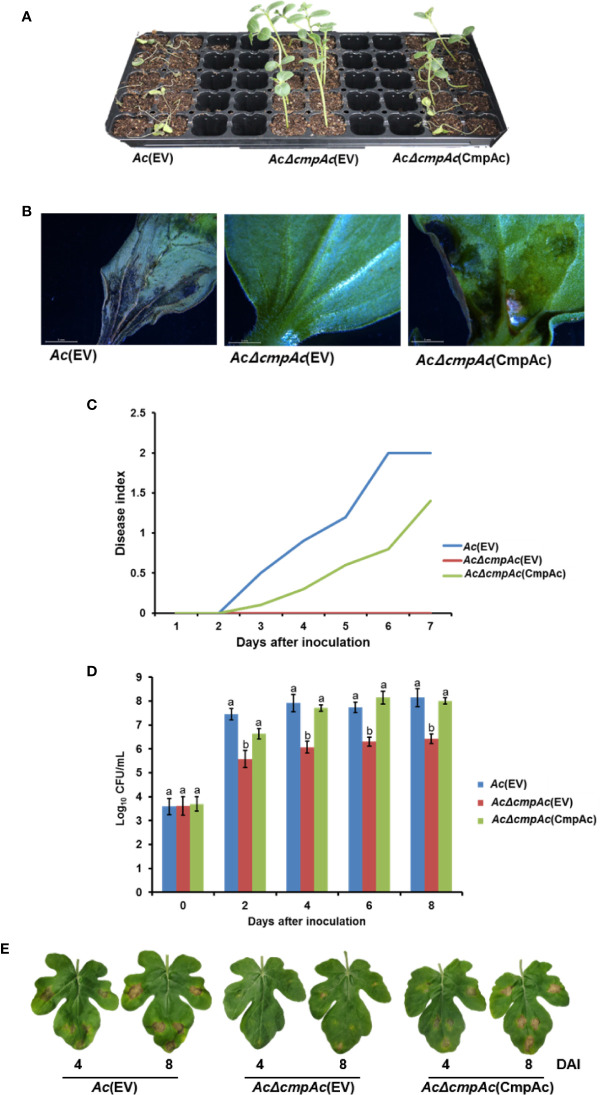
Pathogenicity assay for *Ac*(EV), *AcΔcmpAc*(EV), and *AcΔcmpAc*(CmpAc) using seed-germinated and infiltration inoculation method on watermelon. **(A)** The seed-germinated inoculation results in watermelon seedlings. The photographs were taken at 7 days after inoculation. **(B)** The midrib of infected leaves observed through a stereoscopic microscope. The observations were conducted 7 days after inoculation. **(C)** Disease index variation in the inoculated seedlings during 7 days after inoculation. The disease index is [Normal(numbers of plants)× 0+Spot(numbers of plants)×*1+Wilt(numbers of plants)×2]/Total(numbers of plants). **(D)** The bacterial population in the infected leaves for 8 days after inoculation determined by the colony counting method. The *Ac* strain suspension was adjusted to an O.D._600nm_ of 10^5^ in 10 mM MgCl_2_, and then inoculated into first and second true leaves in 2-week-old watermelon seedlings using a needleless syringe. Different letters on error bars in the graph represent statistically significant differences by ANOVA (p < 0.05), Tukey HSD^ab^. Error bars indicate standard errors of means. The graph was generated from seven independent experiments with three biological replicates. **(E)** The infected watermelon true leaf of each strain. The photographs were taken at 4 and 8 days after inoculation.

To identify whether CmpAc is involved in the virulence of *Ac*, pathogenicity assays using the seed-germinated inoculation and infiltration methods were conducted. *Ac*(EV) and *AcΔcmpAc*(EV), which are *Ac* and *AcΔcmpAc* strains carrying an empty vector, respectively, were used for the assay. In addition, we tested the virulence of the complemented strain, *AcΔcmpAc*(CmpAc), which is *AcΔcmpAc* carrying the CmpAc gene on pBBR1MCS5 vector. As shown in **Figures 2A, B**, seeds infected by *AcΔcmpAc*(EV) did not show any symptoms. However, *Ac*(EV) displayed typical wilt symptoms, and the complemented strain showed spots and some wilts. The disease severity by *AcΔcmpAc*(EV) was 0, while that by *Ac*(EV) was 2 at 7 days after inoculation (**Figure 2C**). The disease severity by *AcΔcmpAc*(CmpAc) was partially restored to the level of *Ac*(EV) at 1.4 (**Figure 2C**). The infiltration on leaves of watermelon showed similar patterns compared to the germinated-seed method (**Figures 2D, E**). Leaves infiltrated by *Ac*(EV) turned dark and wilted, but those by the mutant did not show any symptoms. *AcΔcmpAc*(CmpAc) showed similar results as *Ac*(EV). The bacterial population of *AcΔcmpAc*(EV) in infected leaves was markedly lower than those of *Ac*(EV) and *AcΔcmpAc*(CmpAc) at 2, 4, 6, and 8 days after inoculation. These data indicate that CmpAc is required for the virulence of *Ac*.

### CmpAc Mutant Is a Phenylalanine Auxotroph

**Figure 3:**
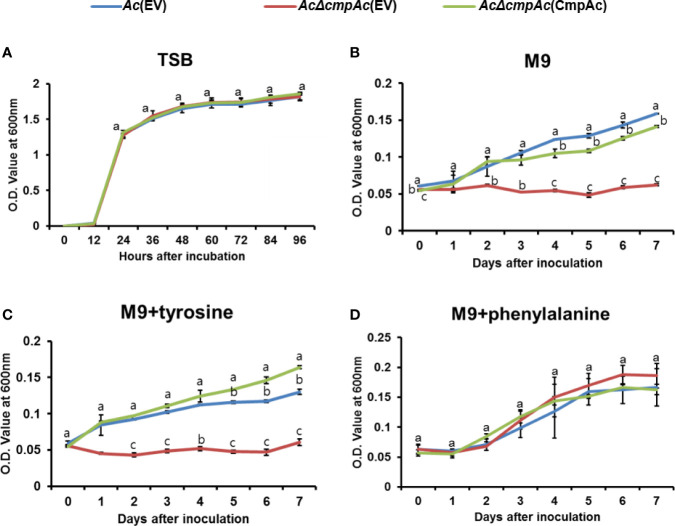
Auxotroph assay. **(A)** Bacterial growth measurement in **(A)** tryptic soy broth (TSB). **(B)** M9 medium without any amino acids. **(C)** M9 medium with 1 mM tyrosine. **(D)** M9 medium with 1 mM phenylalanine. The O.D. value at 600 nm **(A)** for 96 h with 12-h intervals and **(B–D)** for 7 days with 24-h intervals. Error bars indicate standard deviations. The different letters indicate statistically significant differences by ANOVA with Tukey HSD^ab^ (P < 0.05).

In the shikimate pathway, chorismate mutase is responsible for synthesizing both phenylalanine and tyrosine, while prephenate dehydratase is essential for phenylalanine, but not tyrosine. Therefore, the growth of *Ac* strains in the presence of phenylalanine or tyrosine was investigated (**Figure 3**). Firstly, the bacterial growth of the three strains was examined in a rich medium, and all three strains showed similar growth patterns (**Figure 3A**), indicating that CmpAc is not involved in the reproduction of *Ac*. In M9 medium, *AcΔcmpAc*(EV) did not grow without any amino acids (**Figure 3B**). On the other hand, *Ac*(EV) and *AcΔcmpAc*(CmpAc) grew well. In the presence of tyrosine, the growth of the three strains was comparable to those in M9 (**Figure 3C**). Strikingly, the growth of *AcΔcmpAc*(EV) was restored to the level of *Ac*(EV) in M9 with phenylalanine (**Figure 3D**). These results demonstrate that *AcΔcmpAc*(EV) is an auxotroph for phenylalanine, but not tyrosine.

### Proteomic Analysis

**Figure 4:**
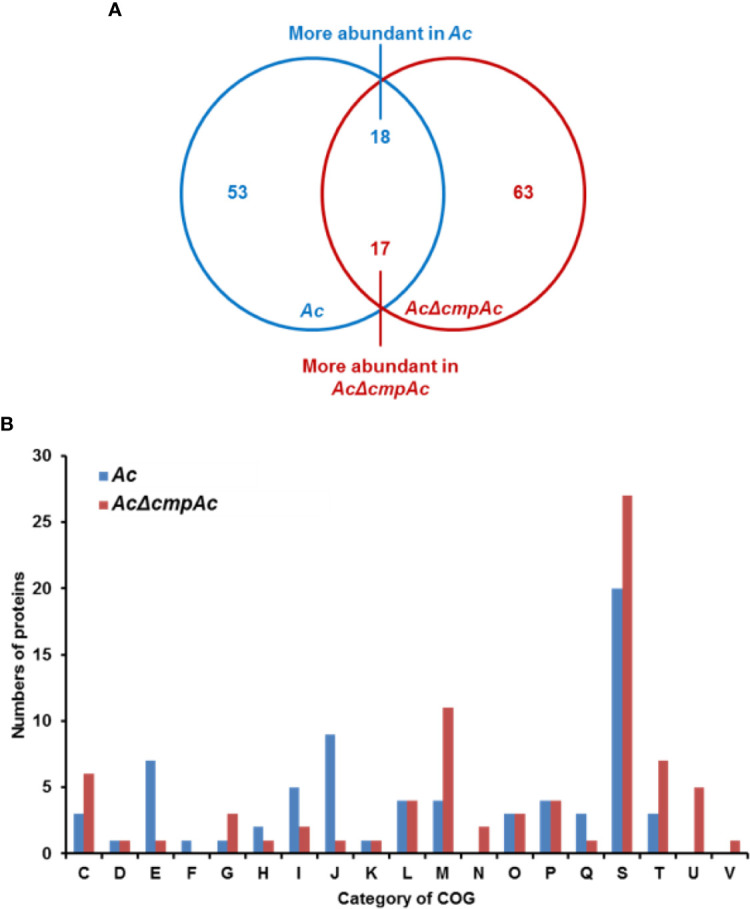
Comparative proteomic analysis in *Ac* and *AcΔcmpAc*. **(A)** The Venn diagram of 71 and 80 proteins whose abundance was altered. Fifty-three and 63 proteins were uniquely found in *Ac* and *AcΔcmpAc*, respectively. Eighteen and 17 proteins were differentially (>2 fold) enriched in *Ac* and *AcΔcmpAc*, respectively. **(B)** Clusters of orthologous groups classification of differentially abundant proteins (>2 fold) from the proteomic analysis. C, Energy production and conversion; D, Cell cycle control and mitosis; E, Amino acid metabolism and transport; F, Nucleotide metabolism and transport; G, Carbohydrate metabolism and transport; H, Coenzyme metabolism; I, Lipid metabolism; J, Translation; K, Transcription; L, Replication and repair; M, Cell wall/membrane/envelop biogenesis; N, Cell motility; O, Post-translational modification, protein turnover, chaperone functions; P, Inorganic ion transport and metabolism; Q, Secondary structure; S, Function unknown; T, Signal transduction; U, Intracellular trafficking and secretion; V, Defense mechanisms.

To further predict the biological or cellular processes affected by CmpAc, the comparative proteomic analysis combined with COG classification was carried out using *Ac* and *AcΔcmpAc* strains. Through LC-MS/MS, 913 and 872 proteins were commonly detected in three biological replicates of *Ac* and *AcΔcmpAc*, respectively (**Supplementary Table 2**), and these proteins were subjected to a comparative proteomic analysis. In the detected proteins, 53 proteins were only detected in *Ac*, and 63 proteins were uniquely found in *AcΔcmpAc* (**Figure 4A**). Additionally, 18 and 17 proteins were differentially (over 2-fold) abundant in *Ac* and *AcΔcmpAc*, respectively (**Figure 4A** and **Supplementary Figure 1**). These proteins were categorized using COG analysis. Proteins belonging to group E (Amino Acid metabolism and transport), I (Lipid metabolism), and J (Translation) were abundantly detected in *Ac* compared to that in *AcΔcmpAc* (**Figure 4B** and **Supplementary Table 3**). In addition, proteins categorized in group C (Energy production and conversion), M (Cell wall/membrane/envelop biogenesis), T (Signal transduction), and U (Intracellular trafficking and secretion) were highly found in *AcΔcmpAc* (**Figure 4B** and **Supplementary Table 4**). Among all the categories in COG, the group M including penicillin-binding protein showed the highest numbers of differentially abundant proteins. In addition, porins, ABC transporters, and methyl-accepting chemotaxis proteins were detected in the comparative proteomic analysis (**Supplementary Tables 3**, **4**). These data suggest that CmpAc may be related to cell membrane/wall integrity.

### CmpAc Is Associated With Biofilm Formation

**Figure 5:**
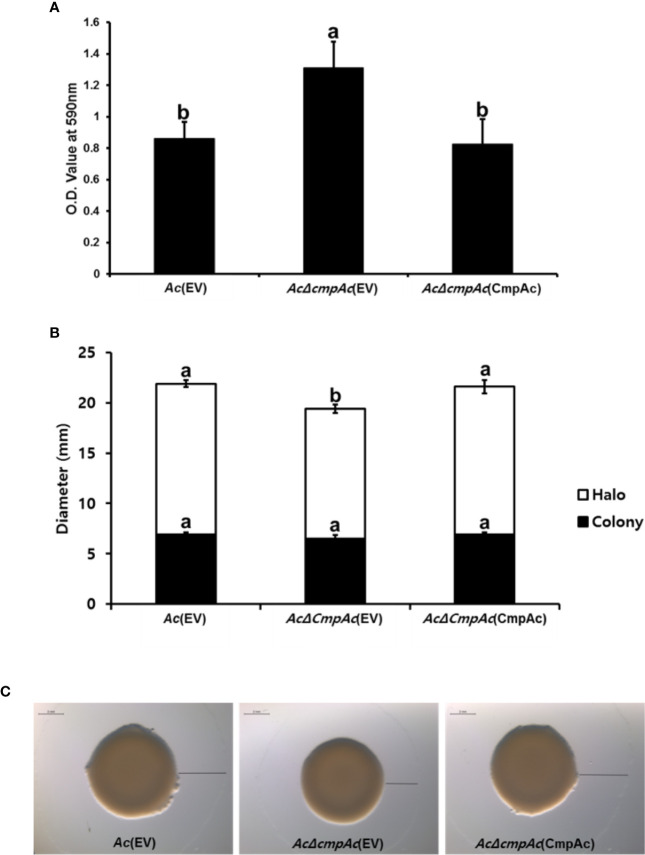
Biofilm formation and twitching motility halo production of *Ac*(EV), *AcΔcmpAc*(EV), and *AcΔcmpAc*(CmpAc). **(A)** Each strain was incubated in a polyvinyl chloride 96-well plate for 2 days. For the quantification, the biofilm was stained with 0.1% crystal violet. The stained biofilm was eluted using 95% ethanol, and the absorbance was measured by UV spectrophotometer at 590 nm. **(B)** Colony and halo production. The black bars indicate the colony diameter. The white bars show the diameter of the twitching halos produced. **(C)** Observation of colony and twitching halo production using a stereoscopic microscope. Black lines indicate the twitching halo diameter. Scale bar, 2 mm. Error bars indicate standard deviations. The different letters indicated statistically significant differences by ANOVA with Tukey HSD^ab^ (P < 0.05).

Our proteomics analysis revealed that the abundance of proteins related to the secretion of polysaccharides was altered. It is well known that the polysaccharide is one of the major components of biofilm in bacteria (Vu et al., 2009). Therefore, we hypothesized that CmpAc might have effects on biofilm formation. The biofilm formation of *Ac* strains was tested (**Figure 5A**) using a 96-well PVC microplate assay. Interestingly, the ability to form biofilm in *AcΔcmpAc*(EV) was enhanced (over 1.5-fold) compared to that in *Ac*(EV). In addition, *AcΔcmpAc*(CmpAc) showed a similar level of biofilm formation as *Ac*(EV). These data indicate that CmpAc is associated with biofilm formation in *Ac*.

### *AcΔcmpAc*(EV) Displayed Reduced Twitching Halos

Proteins associated with pili functions were found in the comparative proteomic analysis. Pili are known to be involved in the twitching motility in bacteria (Mattick, 2002). Thus, the twitching motility assay was conducted in TSA with 0.5% agar, and the diameters of bacterial colonies and twitching halos were measured (**Figures 5B, C**). The colony diameters of *Ac*(EV), *AcΔcmpAc*(EV), and *AcΔcmpAc*(CmpAc) were 6.9, 6.5, and 6.9 mm, respectively (**Figures 5B, C**), indicating that the expansion of the colony was not different in the three strains. The shapes of the marginal sides of colonies of the three strains were also similar (Data not shown). Interestingly, the twitching halos of *AcΔcmpAc*(EV) were reduced (12.9 mm) compared with those of *Ac*(EV) (15 mm). The diameter of the twitching halo in *AcΔcmpAc*(CmpAc) was comparable to that in *Ac*(EV). These results indicate that the functions of CmpAc are related to the production of twitching halos.

### CmpAc Is Involved in Stress Tolerance

**Figure 6:**
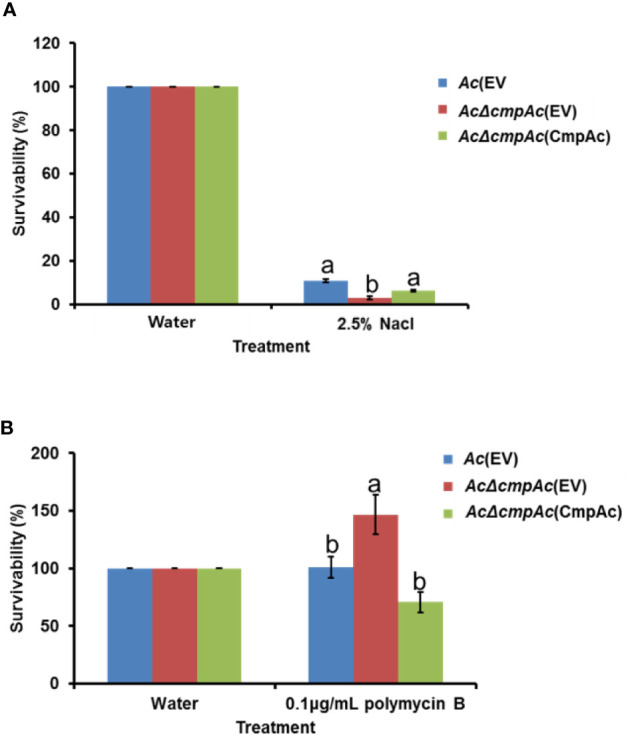
Tolerance assay for osmotic stress and antibiotics. Tolerance was determined in the presence of **(A)** 2.5% NaCl for 10 min or **(B)** 0.1 μg/ml polymyxin B for 2 h. The numbers of recovered bacterial cells were counted by the colony counting method. The survivability was calculated based on the number of recovered cells post water treatment (control) compared to that post the stress treatment. The different letters indicated statistically significant differences by ANOVA with Tukey HSD^ab^ (P < 0.05). Error bars indicate standard errors of means.

In the comparative proteomic analysis, the abundance of proteins related to cell wall/membrane/envelope biosynthesis was mostly affected. Previous studies reported that bacterial cell wall/membrane/envelope is associated with functions for protecting bacteria against the external environment (Hews et al., 2019; Park et al., 2019). Therefore, the tolerance of *AcΔcmpAc*(EV) against two external stresses was tested. Firstly, NaCl was used as an agent for osmotic stress (**Figure 6A**). In the presence of 2.5% NaCl, *Ac*(EV) showed 10.9% survivability, while *AcΔcmpAc*(EV) showed only 2.9% survivability. The survivability of *AcΔcmpAc*(CmpAc) was restored to the level of *Ac*(EV). Unexpectedly, in the supplementation of polymyxin B (0.1 µg/ml), which is a β–lactam antibiotics and has effects on membrane permeability, the survivability of *AcΔcmpAc*(EV) was enhanced (approximately, 1.5-fold) compared with that of *Ac*(EV) (**Figure 6B**). *AcΔcmpAc*(CmpAc) displayed comparable survivability to *Ac*(EV). These data indicate that CmpAc is associated with tolerance to environmental stresses.

## Discussion

It is generally known that the chorismate mutase gene is involved in virulence in both eukaryotic and prokaryotic microorganisms. For example, the inactivation of chorismate mutase in *Ustilago maydis* showed reduced virulence (Djamei and Kahmann, 2012). In contrast, the chorismate mutase knock out of *X. oryzae* pv. *oryzae* XKK.12 was more virulent than the wild-type strain in rice (Degrassi et al., 2010). In our study, we showed that the CmpAc is required for virulence of *Ac* using two different inoculation assays. But, there was no difference in bacterial growth in rich media, indicating that reduced virulence was not due to bacterial multiplication or reproduction. In addition to virulence, proteins required for the biosynthesis of aromatic amino acids are also involved in other mechanisms in bacteria. For example, *aroA* and *aroB* in the shikimate pathway are related to virulence, pigment production, and tolerance to UV stress in *Burkholderia glumae* (Karki and Ham, 2014). Felgner et al. reported that the auxotrophic mutant for aromatic amino acids in *Salmonella enterica* serovar Typhimurium showed the alterations in virulence, the lipid/amino acid metabolisms, sensitivity to penicillin, flagellin phase variation, motility and expression of the virulence-associated genes using transcriptomic and metabolic analyses as well as phenotypic assays (Felgner et al., 2016). In an agreement these studies, we also demonstrated that CmpAc is involved in virulence, phenylalanine biosynthesis, and other mechanisms, including biofilm formation, twitching motility, and stress tolerance by the comparative analysis and phenotypic observation. Thus, it suggests that enzymes, which are required for the biosynthesis of the primary metabolite, including CmpAc may be associated with diverse cellular functions in bacteria.

Chorismate mutase is a key enzyme of the shikimate pathway. Chorismate can be catalyzed by chorismate mutase to produce prephenate which is a branch point for the biosynthesis of tyrosine and phenylalanine (Kast et al., 2000). Prephenate is a substrate of two different enzymes: prephenate dehydratase and prephenate aminotransferase, which are responsible for phenylalanine and tyrosine, respectively (Tzin and Galili, 2010). Interestingly, some bacterial chorismate mutases possess two domains: chorismate mutase and prephenate dehydratase. These bifunctional proteins are involved in the production of phenylalanine, but not tyrosine because prephenate dehydratase is required for the production of phenylalanine (Duggleby et al., 1978; Zhang et al., 1998). In an agreement with these studies, our sequence analysis revealed that the chorismate mutase in *Ac* also contains two putative domains for chorismate mutase and prephenate dehydratase. We further demonstrated that CmpAc is indispensable for biosynthesis of phenylalanine, but not tyrosine in an auxotrophic assay. Interestingly, *Ac* strain KACC17005 possesses another putative chorismate mutase (ATG95797), containing only the chorismate mutase domain (Park et al., 2017). Although we did not test the functions of ATG95797 in this study, the protein may be responsible for the biosynthesis of tyrosine in the shikimate pathway in *Ac*.

Biofilm formation is one of the key elements for bacterial virulence (Davey and O’toole, 2000). Interestingly, *AcΔcmpAc*(EV) showed higher biofilm formation but was less virulent compared to other strains. According to a previous report, impaired capsular polysaccharides in *Streptococcus pneumonia* enhanced the biofilm formation but reduced the virulence (Qin et al., 2013). The proteomics analysis identified that an abundance of proteins involved in cell wall/membrane/envelop biogenesis was altered. In addition, proteins classified in the group U (intracellular trafficking and secretion) were detected only in *AcΔcmpAc*. These results implicate that impaired CmpAc in *Ac* may alter membrane integrity and/or secretion systems. Therefore, it can be postulated that the abnormal cell membranes may be weakened and are easily broken during the protein extraction steps, and hence, proteins related to cell wall/membrane/envelop biogenesis and intracellular trafficking and secretion were abundantly found in the comparative proteomic analysis. Similar to *S. pneumonia* (Qin et al., 2013), it is also hypothesized that the weakened cell membrane/wall may be responsible for the higher biofilm formation observed in *AcΔcmpAc*(EV) compared with other strains.

Biofilm formation is closely related to β-lactamase production in *Pseudomonas aeruginosa* (Heydari and Eftekhar, 2015), postulating that biofilm formation has a positive role for tolerance to β-lactams like polymyxin B. In addition, cell envelope synthesis and modification in *Vibrio cholera* also modulate tolerance to β-lactam antibiotics (Weaver et al., 2018). Similarly, in our study, *AcΔcmpAc*(EV) showed enhanced biofilm formation as well as tolerance to polymyxin B. Moreover, the mutant was less tolerant to osmotic stress compared with *Ac*(EV). In addition to the proteomic analysis, these results also support that CmpAc is associated with cell membrane/wall/envelope biosynthesis. Twitching motility rather than flagella-dependent motility is a crucial mechanism for bacterial movement in *Ac* (Bahar et al., 2010). It is also known that twitching motility in *Ac* is required for optimal virulence (Rosenberg et al., 2018). In *X. oryzae* pv. *oryzae*, the knockout mutant of outer membrane porin, has irregular shape twitching motility and an alteration in stress tolerance (Bae et al., 2018). Likewise, in this study, we showed that *AcΔcmpAc*(EV) reduced the production of twitching halo, which may contribute to the virulence of the group II *Ac* strain KACC17005.

In summary, we demonstrated that CmpAc, which is a putative bifunctional chorismate mutase/prephenate dehydratase, is related to the biosynthesis of phenylalanine, biofilm formation, twitching motility, and tolerance of osmotic stress and antibiotics, which may contribute to the virulence of *Ac*. Several enzymes, including chorismate mutase, which are involved in the production of amino acids, have been thought as potential targets for developing virulence inhibitors (Monti et al., 2016; Khanapur et al., 2017). Therefore, this study provides valuable information on an uncharacterized virulence factor, which could also be a potential target for anti-virulence reagent to control BFB.

## Data Availability Statement

The mass spectrometry proteomics data have been deposited to the ProteomeXchange Consortium *via* the PRIDE (Perez-Riverol et al., 2019) partner repository with the dataset identifier PXD019444.

## Author Contributions

S**-**WH conceived the study. S**-**WH and MK designed the experiments. MK, JL, and LH conducted the experiments. MK and S-WH analyzed the data and prepared the manuscript. All authors contributed to the article and approved the submitted version.

## Funding

This work was supported by the Next-Generation BioGreen 21 Program (PJ01328901) of the Rural Development Administration and the National Research Foundation of Korea (NRF) grant funded by the Korea government (MSIT) (No. NRF-2020R1A2C1013040), Republic of Korea (to S-WH). This research was supported by the Chung-Ang University Graduate Research Scholarship in 2020 (to LH).

## Conflict of Interest

The authors declare that the research was conducted in the absence of any commercial or financial relationships that could be construed as a potential conflict of interest.

## Supplementary Material

Supplementary Figure 1 Differentially abundant (>2 fold) proteins detected in *Ac* and *AcΔcmpAc* strains. Normalized peptide spectral matches of proteins detected in *AcΔcmpAc* (Red) were comparted with these in *Ac* (Blue). Error bars indicate standard deviations. The abundance of all proteins was statistically different (P < 0.05).
