# Supplementary figures and images for: Putative Bifunctional Chorismate Mutase/Prephenate Dehydratase Contributes to the Virulence of Acidovorax citrulli
Source: Front Plant Sci. 2020 Sep 25;11:569552. doi: 10.3389/fpls.2020.569552 (PMC7546022; doi:10.3389/fpls.2020.569552)

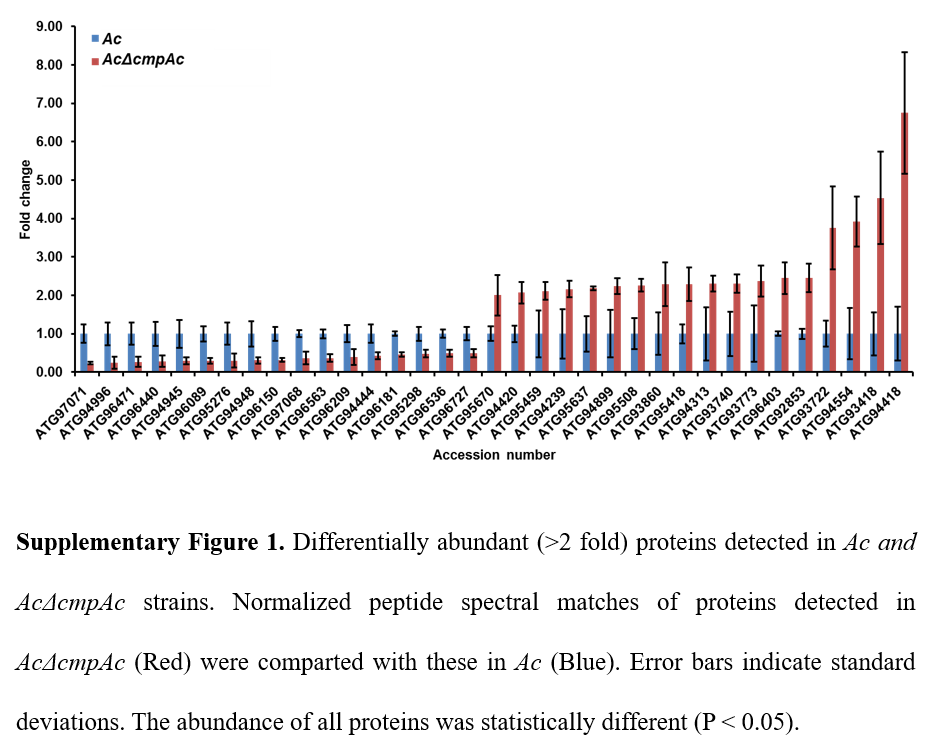

Supplement: Supplementary Figure 1 — Differentially abundant (>2 fold) proteins detected in Ac and AcΔcmpAc strains. Normalized peptide spectral matches of proteins detected in AcΔcmpAc (Red) were comparted with these in Ac (Blue). Error bars indicate standard deviations. The abundance of all proteins was statistically different (P < 0.05). [file Image_1.tif]
