# Supplementary material for: Putative Bifunctional Chorismate Mutase/Prephenate Dehydratase Contributes to the Virulence of Acidovorax citrulli
Source: Front Plant Sci. 2020 Sep 25;11:569552. doi: 10.3389/fpls.2020.569552 (PMC7546022; doi:10.3389/fpls.2020.569552)
Supplement: Supplementary file 2 [file Table_1.docx]

| **Supplementary Table 1. Bacterial strains and plasmids used in this study** | |  |
| --- | --- | --- |
| **Bacterial and plasmid characteristics** |  | **Source or reference** |
|  |  |  |
| ***Escherichia coli*** |  |  |
| **DH5α** | **Strain used for cloning** | **promega** |
| **EC100D** | **Strain used for generate Tn5 inserted plasmid** | **epicentre** |
|  |  |  |
| ***Acidovorax citrulli*** |  |  |
| **KACC17005** | **wild type, complete genome sequence, Rif^r^** |  |
| ***Ac*(EV)** | **wild type carrying the pBBR1-MCS5, Rif^r^,Gm^r^** | **This study** |
| ***AcΔcmpAc*** | **Tn5 insertional knock out mutant in *cmpAc*, Rif^r^, Km^r^** | **This study** |
| ***AcΔcmpAc*(EV)** | ***AcΔcmpAc* carrying pBBR1-MCS5, Rif^r^, Km^r^, Gm^r^** | **This study** |
| ***AcΔcmpAc*(CmpAc)** | **The complemented strain, *AcΔcmpAc* carrying pBBR1-CmpAC, Rif^r^, Km^r^, Gm^r^** | **This study** |
|  |  |  |
| **Plasmid** |  |  |
| **pGem-T easy** | **TA cloning vector, Am^r^** | **Promega** |
| **pGem-CmpAc** | **pGem-T easy contacting 1131-bp DNA fragment (*cmpAc*), Am^r^** | **This study** |
| **pBBR1-MCS5** | **Broad-host-range vector , LacZ promoter, Gm^r^** | **This study** |
| **pBBR1-CmpAc** | **pBBR1-MCS5 carrying *cmpAc* fragment from pGem-CmpAc, Gm^r^** | **This study** |
| **Rif^r^,Gm^r^,Km^r^ and Am^r^ represent resistance to rifampicin, gentamycin, kanamycin and ampicillin, respectively.** | |  |
