# Supplementary material for: Putative Bifunctional Chorismate Mutase/Prephenate Dehydratase Contributes to the Virulence of Acidovorax citrulli
Source: Front Plant Sci. 2020 Sep 25;11:569552. doi: 10.3389/fpls.2020.569552 (PMC7546022; doi:10.3389/fpls.2020.569552)
Supplement: Supplementary file 3 [file Table_2.docx]

| **Supplementary Table 2. Proteins and peptide spectral matches (PSMs) between *Ac* and *AcΔcmpAc*.** | | | | | | | |
| --- | --- | --- | --- | --- | --- | --- | --- |
| **Strain** | **1st** | | **2nd** | | **3rd** | | **shared protein in 3 biological protein** |
|  | **Protein** | **PSM** | **Protein** | **PSM** | **Protein** | **PSM** |  |
| ***Ac*** | **996** | **62,760** | **956** | **62,808** | **976** | **62,797** | **913** |
| ***AcΔcmpAc*** | **922** | **66,308** | **1011** | **65,984** | **1027** | **65,944** | **872** |
